# Organosilicon Compounds, SILA-409 and SILA-421, as Doxorubicin Resistance-Reversing Agents in Human Colon Cancer Cells

**DOI:** 10.3390/molecules25071654

**Published:** 2020-04-03

**Authors:** Olga Wesołowska, Krystyna Michalak, Maria Błaszczyk, Joseph Molnár, Kamila Środa-Pomianek

**Affiliations:** 1 Department of Biophysics and Neuroscience, Wroclaw Medical University, 50-367 Wroclaw, Poland; 2 Institute of Medical Microbiology and Immunobiology, University of Szeged, 6720 Szeged, Hungary

**Keywords:** organosilicon compounds, SILA-409 (Alis-409), SILA-421 (Alis-421), multidrug resistance (MDR) reversal, ABCB1 (P-glycoprotein), colon cancer

## Abstract

Multidrug resistance (MDR) that occurs in cancer cells constitutes one of the major reasons for chemotherapy failure. The main molecular mechanism of MDR is overexpression of protein transporters from the ATP-binding cassette (ABC) superfamily, such as ABCB1 (multidrug resistance protein 1 (MDR1), P-glycoprotein). At the expense of ATP hydrolysis, ABCB1 pumps a diverse range of substrates (including anticancer drugs) out of the cell, thereby reducing their intracellular concentration. In the present study, the ability of two patented disiloxanes (SILA-409 and SILA-421) to reverse drug resistance in human colon adenocarcinoma cell lines LoVo and LoVo/Dx was investigated. It was demonstrated that both compounds in concentrations of 0.5–1 µM strongly increased the sensitivity of LoVo/Dx cells to doxorubicin. By means of an accumulation test in which rhodamine 123 was used as an ABCB1 substrate analogue, both organosilicon compounds were also shown to inhibit ABCB1 transport activity. The intracellular accumulation of doxorubicin was also increased, and more drug entered the cellular nuclei of resistant cells in the presence of the studied compounds. In conclusion, both SILA-409 and SILA-421 were demonstrated to be effective MDR reversal agents in resistant human colon cancer cells.

## 1. Introduction

Since chemotherapy continues to be a method of choice for the treatment of various types of cancer, any factors that undermine its effectiveness constitute a serious therapeutic issue. In the majority of patients, the initial response to chemotherapy is satisfactory; however, the occurrence of multidrug resistance (MDR) during treatment results in a development of progressive disease [1,2]. Cells displaying the MDR phenotype are no longer vulnerable to cytotoxic actions of many functionally and structurally dissimilar anti-cancer drugs.

Among several mechanisms that may lead to the development of MDR, the overexpression of ATP-binding cassette (ABC) transporters such as ABCB1 (P-glycoprotein, MDR1: multidrug resistance protein 1), ABCC1 (MRP1: multidrug resistance-associated protein 1), and ABCG2 (BCRP: breast cancer resistance protein) proteins seems to prevail [3,4]. It was recently reported that non-ABC transporters such as Hedgehog receptor Patched were also engaged in doxorubicin (Dox) efflux and conferred Dox resistance to cancer cells [5,6]. ABCB1 is a transporter that utilizes the energy gained from the hydrolysis of ATP to pump many structurally variable substrates (including xenobiotics and chemotherapeutics) out of the cell [7]. Among the numerous strategies proposed to overcome MDR, the most basic approach is the idea to use the inhibitor of a multidrug transporter (MDR modulator) along with chemotherapy in hopes to increase the intracellular accumulation of an anti-cancer drug and to improve the outcome of the treatment. Although this approach is yet to result in any clinical success [8,9,10], the search for new substances that could serve as clinically applicable MDR modulators is continuously ongoing.

Silicon is a metalloid, which is a group of chemical elements also called semi-metals [11]. It can form chemical bonds with carbon and oxygen, and silicon-containing compounds are widely used in medicine and engineering. Silicone is a synthetic polymer composed of repeating siloxane units. Due to its high biocompatibility and favorable mechanical properties, silicone is used for the production of various medical implants (e.g., breast or testicle), as well as contact lenses [11]. On the other hand, silica materials are applied as controlled drug delivery systems and three-dimensional scaffolds for tissue engineering [11]. The introduction of a silicon atom to a structure usually yields a molecule of higher lipophilicity than its carbon analogue. The increased anti-cancer activity of silicon–indomethacin derivatives was recorded, and it was claimed that the higher lipophilicity of silicon derivatives resulted in their increased uptake by cancer cells [12]. New sila-organosulfur compounds were recently synthesized that were reported to be effective anti-cancer and antibacterial agents [13].

SILA-409 and SILA-421 are water-soluble disiloxanes that were synthesized and patented as putative MDR modulators [14]. They were previously demonstrated to increase fluorescent dye accumulation in human *ABCB1* gene-transfected mouse lymphoma cells and, to much lesser extent, in colon carcinoma Colo320/MDR1-LRP cells, but not in ABCC1 expressing breast cancer (HTB-26/MRP1) and stomach cancer (257P/MDR) cells, which was attributed to their specific interactions with the ABCB1 transporter [15]. The specificity of disiloxanes toward ABCB1 was corroborated in a study by Kars et al. conducted in a model system of breast cancer cells [16]. Recently, the synergism between disiloxanes and amyloid β-protein in the inhibition of ABCB1 transporter activity was reported [17]. In an in vivo study on mice bearing human pancreatic cancer xenografts, the application of SILA-409 resulted in the reduction of tumor growth that was accompanied by increased apoptosis and a reduced level of ABCB1 protein in cancer cells [18]. SILA-421 was found to cause cell-cycle arrest and apoptosis in several non-MDR cancer cell lines [19]. The analysis of the global gene expression profile of HL-60 leukemia cells treated with this compound revealed multiple cellular pathways affected by SILA-421, including DNA replication and transcription processes. Interference with these processes was also attributed to the antimicrobial activity of SILA-409 and SILA-421 compounds [20,21,22], as well as their ability to eliminate resistance-bearing plasmids from *Escherichia coli* strains [23]. Additionally, the weak chemopreventive activity of disiloxanes was reported both in vitro and in vivo [24].

In the present work, the ability of SILA-409 and SILA-421 to reverse Dox resistance in human adenocarcinoma cells LoVo/Dx was demonstrated. The disiloxanes inhibited the transport function of ABCB1 protein both in LoVo/Dx cells and in Madin-Darby Canine Kidney (MDCK) cells expressing human ABCB1 (MDCK-MDR1). The amount of Dox accumulated inside LoVo/Dx cells was also elevated in the presence of studied compounds, and its distribution pattern was changed.

## 2. Results and Discussion

### 2.1. Cytotoxicity of Disiloxanes

**Figure 1:**
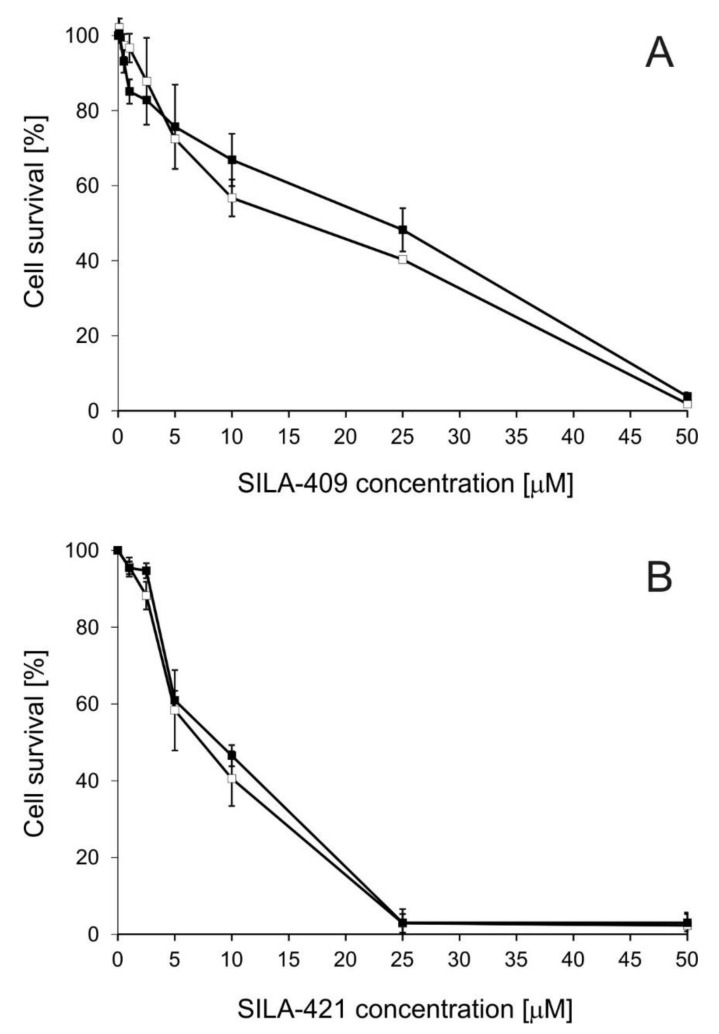
Cytotoxicity of SILA-409 (**A**) and SILA-421 (**B**) to LoVo (open symbols) and LoVo/Dx cells (full symbols). Means of three experiments ± SD are presented.

A human adenocarcinoma cell line sensitive to Dox (LoVo) and its Dox-resistant counterpart (LoVo/Dx) were employed as a model system. It was previously demonstrated that the increased expression of ABCB1 transporter is mainly responsible for the resistance of LoVo/Dx cells [25]. The cytotoxicity of both SILA-409 and SILA-421 to LoVo and LoVo/Dx cells was comparable (Figure 1). Both compounds were strongly cytotoxic to the cells in concentrations above 10 µM. The half maximal inhibitory concentration (IC_50_) values of SILA-409 were 15.6 µM and 24.6 µM for LoVo and LoVo/Dx cells, respectively. For SILA-421, IC_50_ values were 8.4 µM in LoVo cells and 9.2 µM in LoVo/Dx cells. SILA-421 was more toxic than SILA-409, which might be the result of the higher lipophilicity of this compound.

The cytotoxicity of both disiloxanes to MDCK cells and MDCK transfected with human *ABCB1* gene (MDCK-MDR1) was similar (Figure S1, Supplementary Materials). Both compounds in concentrations below 5 µM slightly stimulated cell growth, whereas, in concentrations of 25 µM and higher, <10% of cells survived. IC_50_ values of SILA-409 in MDCK and MDCK-MDR1 cells were 14.2 µM and 13.9 µM, respectively. For SILA-421, the values of 11.6 µM and 12.1 µM were analogously recorded. Similarly to the findings in colon cancer cells, IC_50_ values for SILA-421 were slightly lower than those for SILA-409.

The cytotoxicity of the studied disiloxanes to various cancer cell lines reported previously [15,19] was similar to the results of the present study. The IC_50_ values of SILA-421 analyzed in several various cancer cell lines, as well as in normal cells HEK-293, lay within the range 5–35 µM [19]. In another study, the cytotoxicity of both SILA-409 and SILA-421 was assessed in a panel of multidrug resistant cancer cell lines of different patterns of expression of MDR-associated transporters [15]. IC_50_ values varied from 8–80 μM depending on cell line. In three out of five cell lines studied, SILA-421 was found to be slightly more cytotoxic than SILA-409.

The analysis of the disiloxanes’ chemical structures (Figure S3, Supplementary Materials) and the calculation of their theoretical logP values, performed using the free web tool Molinspiration (https://www.molinspiration.com/cgi-bin/properties), demonstrated that SILA-421 was more lipophilic than SILA-409 (logP values were 5.83 and 9.60 for SILA-409 and SILA-421, respectively). Lipophilicity was observed to positively correlate with the antiproliferative activity of various compounds toward cancer cells [26,27]. However, in other experimental settings, molecular parameters other than lipophilicity were found to affect cytotoxicity to the highest extent [28,29]. Therefore, a larger series of disiloxanes and a characterization of more of their molecular descriptors would be required to determine the effect of lipophilicity on the biological activity of these compounds.

### 2.2. Influence of Disiloxanes on Doxorubicin Cytotoxicity

**Figure 2:**
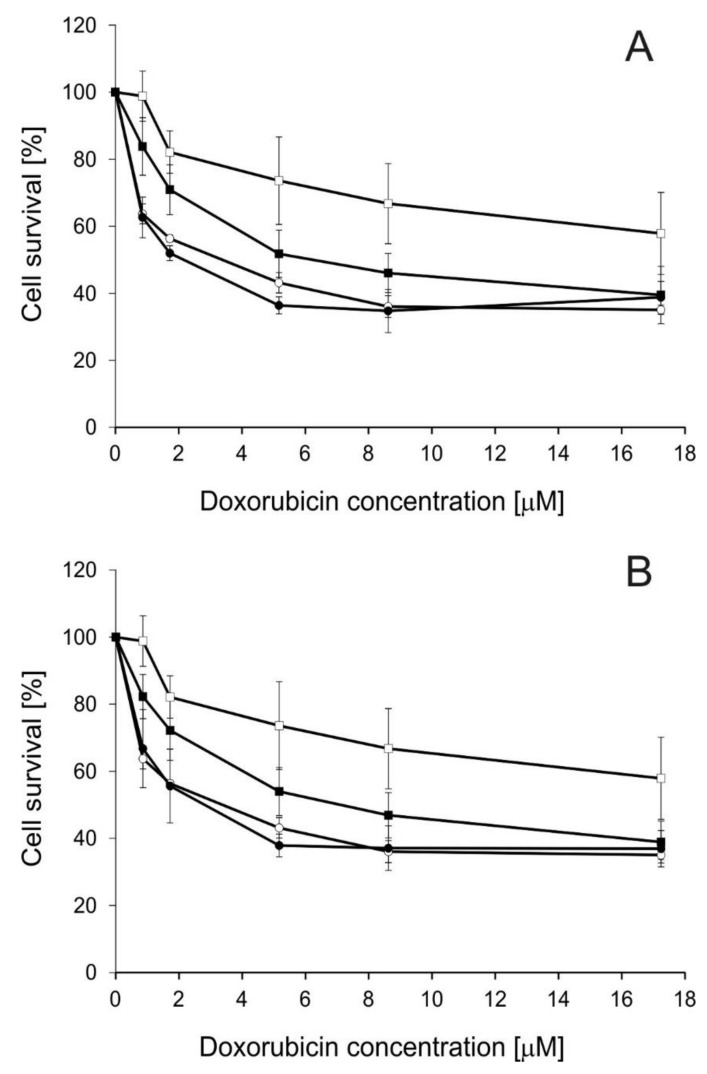
The changes in doxorubicin (Dox) cytotoxicity in LoVo (circles) and LoVo/Dx cells (squares) caused by SILA-409 at 0.5 µM (**A**) and SILA-421 at 1 µM (**B**). Open symbols represent cells treated with Dox only, whereas full symbols represent cells treated with Dox and the modulator. Means of three experiments ± SD are presented.

Next, the influence of the studied modulators on Dox cytotoxicity was investigated (Figure 2). SILA-409 and SILA-421 were applied in concentrations in which they killed <10% of LoVo/Dx cells. The treatment of cells with a combination of either SILA-409 or SILA-421 with Dox significantly reduced the survival rate of resistant colon cancer cells, i.e., the disiloxanes partially reversed Dox resistance. Non-toxic concentrations of SILA-409 and SILA-421 were applied together with doxorubicin. Sensitive LoVo cells were more vulnerable to this anticancer drug (IC_50_ Dox = 4.0 µM) than ABCB1-overexpressing LoVo/Dx cells (IC_50_ Dox = 30.0 µM) [30]. It was demonstrated that both compounds strongly increased the sensitivity of LoVo/Dx cells to doxorubicin without changing the sensitivity of LoVo cells to this drug. The value of IC_50_ for Dox was reduced to 5.7 µM in the presence of SILA-409 at 0.5 µM concentration, and to 6.1 µM in the presence of SILA-421 (1 µM). The isobolographic analysis applied to the obtained results revealed the existence of synergism between Dox and each of the studied disiloxanes (Table 1).

The reduction of Dox cytotoxicity by SILA-409 and SILA 421 was previously observed in human *ABCB1* gene-transfected mouse lymphoma cells (L5718/MDR) and the resistant colon cancer cell line (Colo320/MDR1-LRP) [15]. Synergism between disiloxanes and Dox was observed in these cell lines but not in breast cancer cell lines MCF-7, T47-D, and HTB-26/MRP1. The authors concluded that the presence of a functional ABCB1 transporter was essential for disiloxanes to be able to revert Dox resistance [15]. This pointed to the specific interactions between SILA-409 and SILA-421 and ABCB1 protein. Similar synergistic interactions with appropriate anticancer drugs were also observed for SILA-409 and SILA-421 in MCF-7 cell lines resistant to paclitaxel and docetaxel, but not in the sublines resistant to Dox and vincristine [16].

Dox cytotoxicity was significantly lower (*p* < 0.05) in LoVo/Dx than in LoVo cells as determined by Student’s *t*-test. Significant enhancement (*p* < 0.05) of Dox cytotoxicity was recorded for both SILA-409 and SILA-421in LoVo/Dx cells in the whole concentration range

### 2.3. Intracellular Accumulation of Rhodamine 123 (R123)

**Figure 3:**
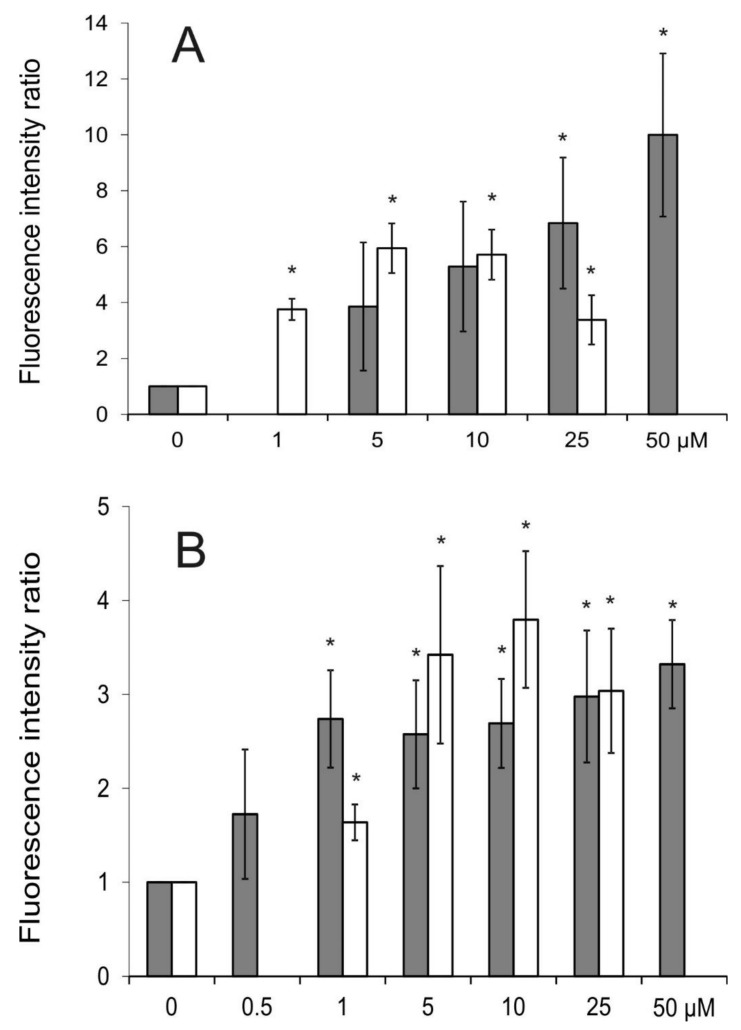
The influence of SILA-409 (gray) and SILA-421 (white) on R123 intracellular accumulation in LoVo/Dx (**A**) and Madin-Darby Canine Kidney-multidrug resistance protein 1 (MDCK-MDR1) cells (**B**). Means ± SD of three experiments are presented. The statistically significant differences from the untreated cells were determined using Student’s *t*-test (* *p* < 0.05).

Rhodamine 123 (R123) is popularly used as a fluorescent reporter substrate of the ABCB1 transporter. Both SILA-409 and SILA-421 caused an increase in R123 accumulation in LoVo/Dx and MDCK-MDR1 cells (Figure 3) in a concentration-dependent manner, which suggested that both compounds were inhibitors of ABCB1 transport activity. SILA-421 seemed to exert its inhibitory action in lower concentrations than SILA-409, which significantly elevated fluorescence intensity ratio (FIR) values only in concentrations above 10 µM in Dox-resistant colon cancer cells.

Molnar et al. [15] also observed increased R123 accumulation in human *ABCB1* gene-transfected mouse lymphoma cells (L5718/MDR) treated with SILA-409 and SILA-421 in concentrations up to 2 μg/mL. Both compounds demonstrated similar activity in this respect. They were, however, unable to affect R123 accumulation in sensitive breast and prostate cancer cells and it was again concluded that the activity of disiloxanes can only be observed in cell lines that express a functional ABCB1 transporter [15]. In the study of Kars et al. [16], both disiloxanes were demonstrated to increase R123 accumulation in a series of breast cancer cell lines resistant to paclitaxel, docetaxel, Dox, and vincristine. In contrast to our findings, SILA-409 was observed to be more active then SILA-421 in this experimental setting.

### 2.4. Intracellular Accumulation of Doxorubicin

**Figure 4:**
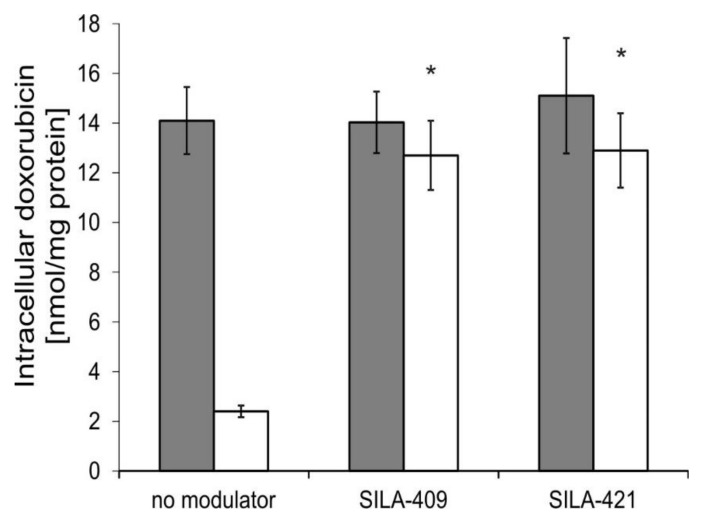
Intracellular doxorubicin (Dox) accumulation in LoVo (gray bars) and LoVo/Dx cells (white bars) treated with SILA-409 and SILA-421 at 5 µM concentration. Means ± SD of three experiments are presented. The statistically significant differences between untreated and disiloxane-treated cells were determined using Student’s *t*-test (* *p* < 0.05).

Accumulation of Dox itself by human colon cancer cells was also investigated. As presented in Figure 4, drug-sensitive LoVo cells accumulated more drug than Dox-resistant LoVo/Dx cells. The treatment of cells by disiloxanes at a concentration of 5 µM resulted in a significant increase in Dox accumulation by LoVo/Dx cells with apparently no effect observed in LoVo cells.

**Figure 5:**
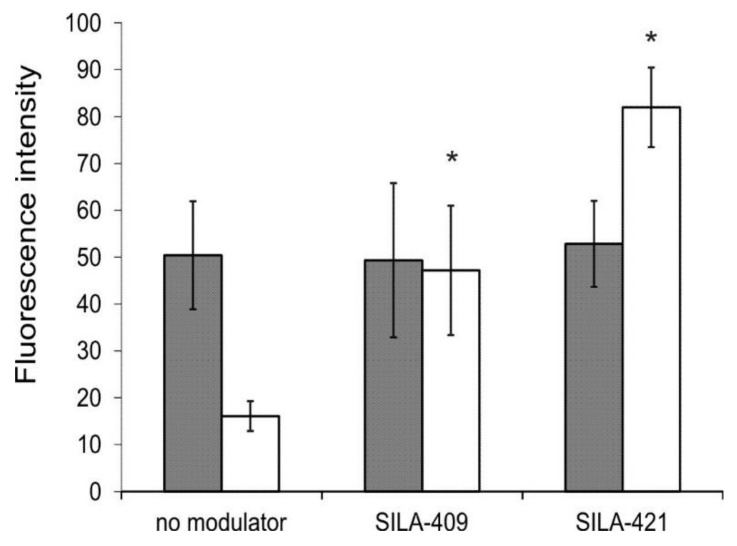
Intensity of intracellular fluorescence of Dox, measured by ImageJ software, in LoVo (gray bars) and LoVo/Dx cells (white bars) treated with 5 µM of SILA-409 and SILA-421 is presented as the mean fluorescence values ± SD measured in 20 representative cells. The statistically significant differences from the untreated cells were determined using Student’s *t*-test (* *p* < 0.05).

Since Dox is characterized by strong intrinsic fluorescence, its intracellular accumulation may also be examined directly by means of fluorescence microscopy. Again, LoVo cells accumulated more Dox than LoVo/Dx cells (Figure 5 and Figure S2, Supplementary Materials), and the application of disiloxanes at a concentration of 5 µM resulted in an increase in accumulation of the drug in Dox-resistant cells but not in the sensitive ones. SILA-421 was more effective in this respect than SILA-409. The intracellular distribution of Dox was also affected by organosilicon compounds. Dox that was excluded from the nuclei of untreated resistant cells was demonstrated to localize within these organelles in modulator-treated LoVo/Dx cells.

According to our best knowledge, the influence of SILA-409 and SILA-421 on Dox accumulation and cellular localization was not previously studied. The pattern of changes in Dox accumulation caused by disiloxanes was similar to the changes caused by verapamil (a well-known inhibitor of ABCB1 protein) observed previously in the same colon cancer cell lines as those used in the present work [25].

### 2.5. Expression of ABCB1 Transporter

**Figure 6:**
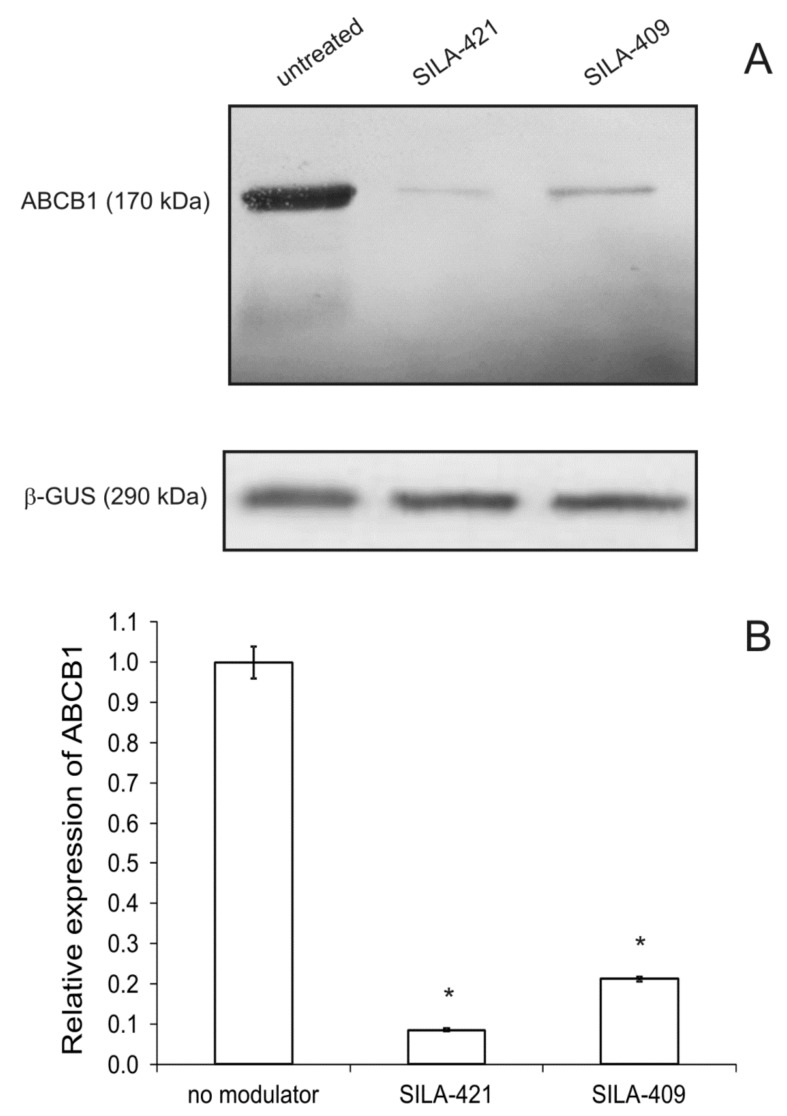
Western blot analysis of ATP-binding cassette B1 (ABCB1) protein level (**A**) in LoVo/Dx cells cultured with 0.5 μM SILA-409 and SILA-421 for 48 h. The molecular masses of the proteins are indicated on the left side of the gel. β-Glucuronidase (β-GUS) was used as a reference protein. The relative level of ABCB1 expression (**B**) was normalized to the control derived from non-treated LoVo/Dx cells. The results of three experiments ± SD are presented. The statistically significant differences from the untreated controls were determined using Student’s *t*-test (* *p* < 0.05).

Additionally, the influence of disiloxanes on ABCB1 transporter expression was analyzed by Western blotting (Figure 6). Both SILA-409 and SILA-421 significantly decreased ABCB1 protein level in LoVo/Dx cells during 48 h of treatment. SILA-421 turned out to be more active in this respect in comparison with SILA-409. No effect of organosilicon compounds on ABCB1 protein level was noted during 60 min of treatment with modulators (data not shown).

The downregulation of ABCB1 expression was previously demonstrated using immunohistochemical methods in pancreatic tumor samples isolated from mice treated with SILA-409 [18]. On the other hand, no influence of disiloxanes (tested in concentrations of 380–500 μg/mL) on *ABCB1* expression level was detected in mouse T-lymphoma cells transfected with human *ABCB1* [15].

## 3. Materials and Methods

### 3.1. Chemicals

Orgasnosilicon compounds 1,3-dimethyl-1,3-bis(4-fluorophenyl)-1,3-bis(3-morpholino-propyl)disiloxan dihydrochloride (SILA-409) and 1,3-dimethyl-1,3-bis(4-fluorophenyl)-1,3- bis{3-[1(4-butylpiperazinyl)]-propyl}-disiloxan tetrahydrochloride (SILA-421) were synthesized and patented [14]. Their chemical structures are presented in Figure S3 (Supplementary Materials). Stock solutions of organosilicon compounds were prepared in dimethyl sulfoxide (DMSO). Sulforhodamine B (SRB), rhodamine 123 (R123), and doxorubicin (Dox) were obtained from Sigma-Aldrich (Poznan, Poland) and dissolved in water.

### 3.2. Cell Culture

Human colorectal adenocarcinoma cell line, LoVo, and its resistant subline, LoVo/Dx, obtained by prolonged exposure to Dox [31], were obtained from the Institute of Immunology and Experimental Therapy of Polish Academy of Science (Wroclaw, Poland). Cultivation conditions were Ham’s F12 medium (with the addition of 10% fetal bovine serum, l-glutamine, antibiotics, and, in the case of LoVo/Dx cells, Dox at 100 ng/mL), at 37 °C and 5% CO_2_.

Madin-Darby Canine Kidney cells (MDCK) and MDCK cells expressing human ABCB1 (MDCK-MDR1) [32] were purchased from the Netherlands Cancer Institute (NKI-AVL, Amsterdam, the Netherlands). The cells were cultured in DMEM medium supplemented with 10% fetal bovine serum, l-glutamine, and antibiotics at 37 °C and 5% CO_2_.

### 3.3. Cell Viability Assay

The SRB assay [33] with minor modifications was used for the estimation of the effect of the studied compounds on cell growth. Cells were seeded in 96-well flat-bottom microtiter plates in 75 μL of medium and allowed to attach (60 min, 37 °C). Then, 75 μL of medium containing an amount of the studied compounds (such that a desired compound concentration was obtained in a final sample volume) was added to each well, with the exception of the control wells, which contained medium only. The culture plates were then incubated for 48 h at 37 °C. The further procedure was carried out as previously described [34]. Cytotoxicity of DMSO to LoVo and LoVo/Dx cells was negligible.

### 3.4. Isobolographic Analysis

Combination index (CI) values were calculated using the CompuSyn software (www.combosyn.com, ComboSyn, Inc., Paramus, USA) according to the classic median-effect equation as described by Chou and Martin [35].

(1) $$CI=\frac{{\left(D\right)}_{1}}{{\left(Dx\right)}_{1}}+\frac{{\left(D\right)}_{2}}{{\left(Dx\right)}_{2}},$$

where (Dx)_1_ is the dose of drug 1 alone that inhibits a system by x%, (Dx)_2_ is the dose of drug 2 alone that inhibits a system by x%, and (D)_1_ + (D)_2_ are doses of drugs 1 and 2 in combination that also inhibit a system by x%.

### 3.5. Accumulation of Rhodamine 123 in Cancer Cells

In order to determine R123 accumulation, the cells were harvested and incubated (300,000 cells/mL) with the appropriate concentration of the studied compound (15 min; 25 °C). Next, R123 (10 μM) was added, and the cells were incubated for 60 min at 37 °C. After centrifugation, the samples were washed twice with ice-cold phosphate-buffered saline (PBS) and dissolved in lysis buffer (20 mM Tris-HCl, 0.2% SDS, pH = 7.7). Intracellular fluorescence (λ_ex_ = 485 nm, λ_em_ = 538 nm) was collected with the use of an Infinite M200Pro plate reader (Tecan Instruments, Maennedorf, Switzerland). Based on measured the fluorescence intensity (FL) of the treated and control samples (without modulator), the fluorescence intensity ratio (FIR) was calculated according to the following equation:

(2) $$\mathrm{FIR}=\frac{\left({\mathrm{FL}}_{\mathrm{LoVoDx}\;\mathrm{or}\;\mathrm{MDCK}-\mathrm{MDR}1\;\mathrm{treated}})/({\mathrm{FL}}_{\mathrm{LoVoDx}\;\mathrm{or}\;\mathrm{MDCK}-\mathrm{MDR}1\;\mathrm{control}}\right)}{\left({\mathrm{FL}}_{\mathrm{LoVo}\;\mathrm{or}\;\mathrm{MDCK}\;\mathrm{treated}}\right)/\left({\mathrm{FL}}_{\mathrm{LoVo}\;\mathrm{or}\;\mathrm{MDCK}\;\mathrm{control}}\right)}.$$

### 3.6. Intracellular Accumulation of Doxorubicin

Intracellular Dox accumulation was detected with a fluorimetry based assay as described previously [36]. Briefly, cells were seeded (800,000/well) onto a six-well plate and incubated for 24 h at 37 °C. Then, the cells were incubated in PBS containing Dox (4 µM) and treated with the modulators. After 48 h of incubation, cells were washed twice in ice-cold PBS and detached. Next, cells were centrifuged and lysed. The cellular protein content was determined using the standard method of Bradford reaction [37]. Dox content was measured using an LS-5 spectrofluorimeter (Perkin-Elmer, Beaconsfield, UK). Excitation and emission wavelengths were 475 and 553 nm, respectively. Fluorescence was expressed in ng of Dox per mg of cellular protein with the use of the calibration curve prepared previously.

For fluorescence microscopic experiments, LoVo and LoVo/Dx cells were cultivated on eight-well µ-Slide microscopy chambers (Ibidi, Munich, Germany) for 48 h. For the experiment, a fresh portion of F12 medium was added containing 50 µM Dox (plus 5 µM of the studied compounds in treated samples), and cells were incubated for 60 min at 37 °C. After incubation, the chambers were washed with PBS and with serum- and phenol red-free F12 medium. The images were collected with a Nikon Eclipse TE2000-E microscope. Fluorescence was excited in the range 528–553 nm and collected in the range 578–633 nm.

### 3.7. Expression of ABCB1 Protein

Cell lysates were prepared in ice-cold lysis buffer (1% Triton X-100, 50 mM Hepes, 150 mM NaCl, 1.5 mM MgCl_2_, 1 mM ethylene glycol-bis(β-aminoethyl ether)-*N,N*,*N*′,*N*′-tetraacetic acid (EGTA), 1 mM phenylmethylsulfonyl fluoride (PMSF), 100 mM NaF, 10 mM sodium pyrophosphate, 10 μg/mL aprotinin, and 10% glycerol, pH 7.4). After centrifugation of whole-cell lysates (13,000× *g*, 10 min, 4 °C), the supernatants were taken for analysis. The standard method of Bradford reaction was used to measure protein content (Bradford, 1976). The proteins were subjected to SDS-PAGE, transferred to polyvinylidene difluoride (PVDF) membranes, and detected using primary antibodies in tris-buffered saline with Tween (TBS-T) buffer (0.1% Triton X-100, 50 mM Tris-HCl, 150 mM NaCl, 1, pH 7.4) with 5% bovine serum albumin (BSA). The anti-ABCB1 mouse monoclonal primary antibodies (C494) (Alexis) were used at dilution 1:1000. The level of β-glucuronidase (β-GUS) was also determined as a reference protein (anti-glucuronidase mouse monoclonal antibody, diluted 1:1000, Thermo Scientific). After incubation (overnight at 4 °C), the membranes were washed in TBS-T and incubated with rabbit anti-mouse immunoglobulin G (IgG) secondary antibody conjugated to horseradish peroxidase (HRP) (dilution 1:1000, Thermo Scientific) for 30 min at 4 °C. The membranes were then washed with TBS-T, and the proteins were visualized. The relative amount of protein normalized to the control (non-treated cells) was determined. The optical density of the bands on the electrophoregram was detected with the Image J softwar version 1.43m.

### 3.8. Statistical Analysis

Data represent the means ± standard deviation (SD) of at least three replications. Student’s *t*-test was applied, and *p*-values less than 0.05 were considered to be statistically significant.

## 4. Conclusions

Organosilicon compounds, SILA-409 and SILA-421, were demonstrated to reverse doxorubicin resistance in a human colon cancer cell line. Both compounds were inhibitors of the transport function of ABCB1 protein judging from the increased accumulation of R123 by modulator-treated cells. Intracellular accumulation of Dox was also increased, and more drug entered cellular nuclei in the presence of the studied compounds. SILA-421 demonstrated slightly higher activity than SILA-409. Additionally, the decreased expression of ABCB1 protein after treatment with disiloxanes was recorded. Therefore, the studied compounds acted as resistance-reversing agents via two mechanisms. They were effective inhibitors of the ABCB1 transport function, and its reduction was easily observed after 60 min of disiloxane treatment (e.g., in fluorescence microscopy and R123 accumulation experiments). Moreover, after prolonged treatment with SILA-409 and SILA-421, the reduction of ABCB1 protein level that occurred certainly increased the MDR-reversing potency of these compounds. In conclusion, both SILA-409 and SILA-421 were demonstrated to be effective anti-MDR agents in resistant human colon cancer cells.

## Figures and Tables

**Table 1:** Combination of disiloxanes with Dox against LoVo/Dx cell growth.

Concentration (µM)		Ratio	Combination Index
Dox	SILA-409		
8.62	0.5	17.24:1	0.7772
17.24	0.5	34.48:1	0.6136
	SILA-421		
8.62	1	8.62:1	0.6467
17.24	1	17.24:1	0.5632

Dose and effect data were obtained from the sulforhodamine B (SRB) assay (mean values of three experiments) and subjected to CompuSyn analysis. CI (combination index) values were generated by CompuSyn software. CI = 1 indicates additive effect, CI < 1 indicates synergism, and CI > 1 indicates antagonism.

## Supplementary Materials

The following are available online at https://www.mdpi.com/1420-3049/25/7/1654/s1: Figure S1: Cytotoxicity of SILA-409 (A) and SILA-421 (B) to MDCK (full symbols) and MDCK-MDR1 cells (open symbols). Means of three experiments ± SD are presented; Figure S2: Fluorescence microscopy images illustrating doxorubicin accumulation in LoVo (A) and LoVo/Dx (B) cells treated with 5 µM SILA-409 (C and D for LoVo and LoVo/Dx, respectively) and with 5 µM SILA-421 (E and F). Scale bar is 50 µm. Illumination conditions were the same for all images; Figure S3: Chemical structures of SILA-409 and SILA-421.
