# Supplementary material for: Organosilicon Compounds, SILA-409 and SILA-421, as Doxorubicin Resistance-Reversing Agents in Human Colon Cancer Cells
Source: Molecules. 2020 Apr 3;25(7):1654. doi: 10.3390/molecules25071654 (PMC7181076; doi:10.3390/molecules25071654)
Supplement: Supplementary file 1 [file molecules-25-01654-s001.pdf]

Supplementary Materials

Organosilicon compounds, SILA-409 and SILA-421, as doxorubicin resistance-reversing agents in human colon cancer cells

Olga Wesołowska [1,*], Krystyna Michalak [1], Maria Błaszczyk [1], Joseph Molnár [2] and Kamila Środa-Pomianek [1]

[1] Department of Biophysics and Neuroscience, Wroclaw Medical University, Poland
[2] Institute of Medical Microbiology and Immunobiology, University of Szeged, Hungary
* Correspondence: olga.wesolowska@umed.wroc.pl; Tel.: +48-71-784-14-15

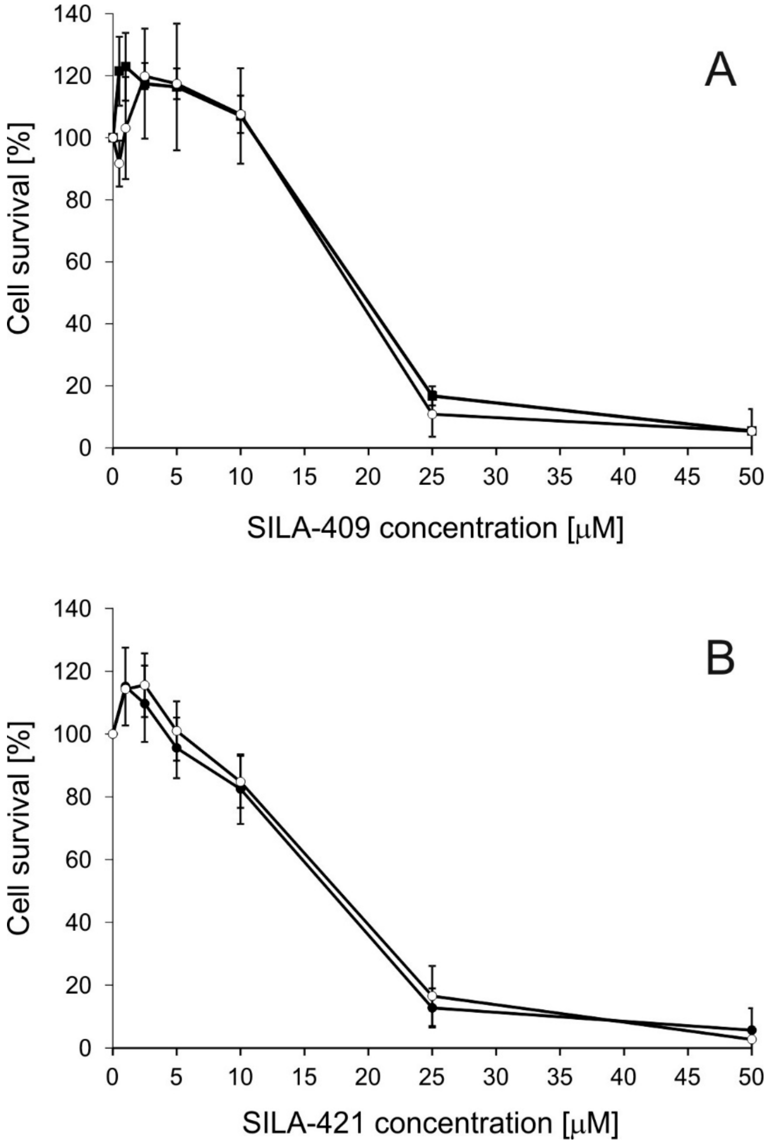

Figure S1. Cytotoxicity of SILA-409 (**A**) and SILA-421 (**B**) to MDCK (full symbols) and MDCK-MDR1 cells (open symbols). Means of three experiments ± SD are presented.

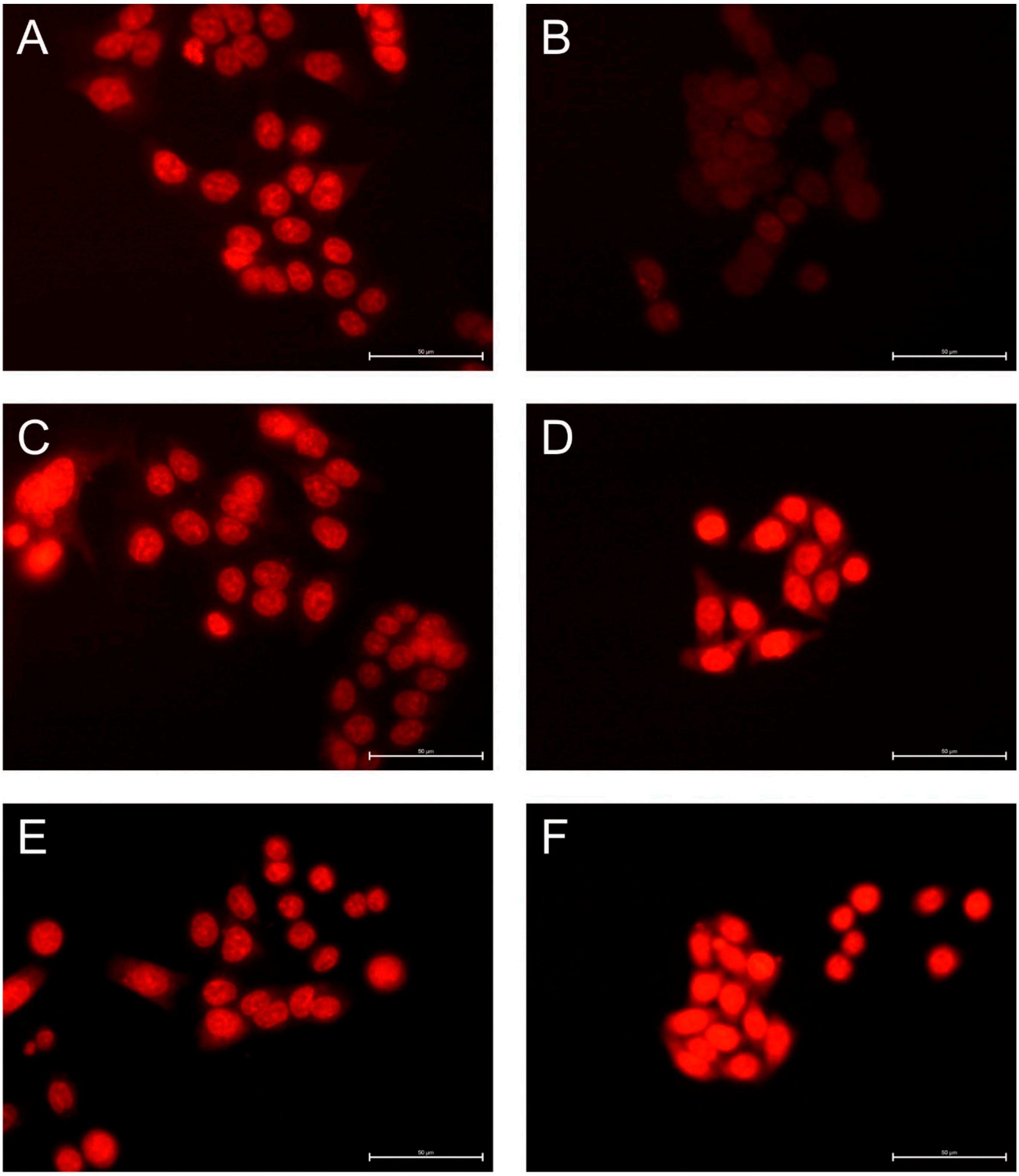

Figure S2. Fluorescence microscopy images illustrating doxorubicin accumulation in LoVo (**A**) and LoVo/Dx (**B**) cells treated with 5 µM SILA-409 (C and D for LoVo and LoVo/Dx, respectively) and with 5 µM SILA-421 (E and F). Scale bar is 50 µm. Illumination conditions were the same for all images.

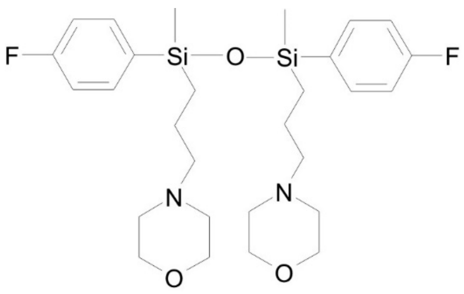

SILA-409

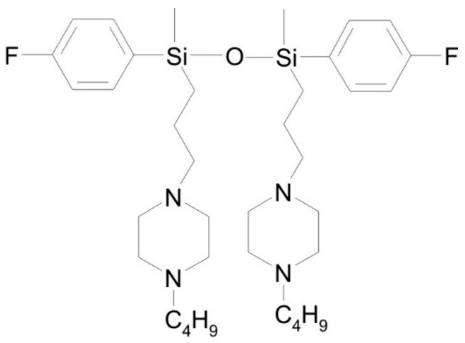

SILA-421

Figure S3. Chemical structures of SILA-409 and SILA-421.
